# The application and value of continuous nursing in patients after coronary artery bypass grafting

**DOI:** 10.1186/s13019-020-01210-2

**Published:** 2020-07-10

**Authors:** Sheng-Huo Zhou, Shu-Ting Huang, Ning Xu, Qiang Chen, Liang-Wang Chen, Yur-Ren Kuo

**Affiliations:** 1 Department of Cardiovascular Surgery, Union Hospital, Fujian Medical University, Fuzhou, 350001 People’s Republic of China; 2 Division of Plastic Surgery, Department of Surgery, Kaohsiung Medical University Hospital, 100 TzYou 1st Rd, Kaohsiung City, 80756 Taiwan

**Keywords:** Continuous nursing, coronary artery bypass grafting, Quality of life

## Abstract

**Objective:**

To investigate the application and value of continuous nursing after coronary artery bypass grafting.

**Methods:**

The clinical data of 62 patients after coronary artery bypass grafting from January 2016 to January 2018 were analyzed retrospectively. According to the nursing mode, the patients were divided into two groups: the continuous nursing group (*n* = 30) and the conventional nursing group (*n* = 32). All patients completed Self-Rating Depression Scale (SDS) and Self-Rating Anxiety Scale (SAS) at admission and 1 year after operation. All patients completed Seattle Angina Pectoris Questionnaire (SAQ) at discharge and 1 year after operation.

**Results:**

All patients were followed up for more than one year. One year after operation, SAQ score in five items in continuous nursing group was significantly better than that in conventional nursing group.(*P* < 0.05) The continuous nursing group exhibited significantly decreased SAS and SDS scores 1 year after surgery compared to the preoperative SAS and SDS scores.(*P* < 0.05) The SAS and SDS scores of the continuous nursing group were significantly better than those of the conventional nursing group 1 year after surgery.(*P* < 0.05) Then incidence rate of chest tightness or chest pain and coronary restenosis in continuous nursing group were significantly less than that in conventional nursing group.(*P* < 0.05).

**Conclusion:**

Continuous nursing improved patient compliance with treatment and reduces the occurrence of complications. The patient also receives proper psychological evaluations, which relieve patient anxiety and depression and improve the quality of life.

## Introduction

Coronary artery disease is a kind of heart disease of myocardial ischaemia, hypoxia or myocardial necrosis caused by organic stenosis or obstruction of the coronary artery, and it is one of the diseases with the highest mortality rates in the world [1, 2]. Coronary artery bypass grafting is an effective method to treat such disease, which can effectively improve the symptoms of myocardial ischaemia [3]. But it is still with a long course and a repeated condition that requires long-term treatment, and needs long-term and repeated treatment after discharge [4]. Patients’ self-management abilities and compliance with treatment decreases may be results in coronary artery restenosis that causes a heavy burden to the family and to society and even endangers the life of patients [5, 6]. Therefore, it is very important to extend high-quality medical service to the patients and their family to understand the compliance behaviour, treatment effect and psychological state of the patients after discharge and provide the patients with medical and psychological guidance at the same time [7–10]. The aim of this study is to explore a better nursing method for patients who have undergone coronary artery bypass grafting by summarizing the experience of continuous nursing in patients after coronary artery bypass grafting at our hospital.

## Materials and methods

The present study was approved by the ethics committee of Fujian Medical University, China and adhered to the tenets of the Declaration of Helsinki. Additionally, all patients signed the consent form before participating in the study.

### Patients

The clinical data of 62 patients after coronary artery bypass grafting from January 2016 to January 2018 were analyzed retrospectively. According to the nursing mode, the patients were divided into two groups: the continuous nursing group (group A, *n* = 30) and the conventional nursing group (group B, *n* = 32). All patients completed the Self-Rating Depression Scale (SDS) and Self-Rating Anxiety Scale (SAS) at admission and 1 year after surgery. All patients completed the Seattle Angina Pectoris Questionnaire (SAQ) at discharge and 1 year after operation. All the patients’ general clinical data are shown in Table 1. Patients met the inclusion criteria if they underwent coronary artery bypass grafting. The following exclusion criteria were used: 1) lack of independent reading ability, 2) lack of communication equipment and difficult communication information, 3) postoperative death, 4) severe disease of other organs or 5) refusal of continuing nursing services and participation in the study.

**Table 1:** Comparison of general data between the two groups

Item	Group A	Group B	*P* value
**Number**	30	32	
**Age (year)**	52.6 ± 8.3	55.6 ± 10.1	0.768
**Man/woman**	19/11	21/11	0.851
**Hypertension**	14	16	0.793
**Diabetes mellitus**	9	7	0.465
**Hyperlipemia**	5	6	0.830
**Smoking**	8	10	0.691
**Alcoholism**	4	4	0.922
**Record of formal schooling**			
**Junior high school and below**	22	23	0.782
**Senior middle school**	5	7	
**Universities and above**	3	2	
**Place of residence: rural / urban**	21/9	22/10	0.915

### Nursing methods

#### Continuing nursing

On the basis of conventional nursing, the method of continuous nursing involved issuing contact cards at discharge. The information on the contact card included basic patient information and the telephone number and WeChat of the attending doctor, attending nurse and head of the department. The nurse instructed the patient to join the WeChat group and provided the patient with instructions on how to use the WeChat function correctly and skilfully after surgery. One team member participated in the online WeChat support group every day from 18:00 to 21:00 h to answer patients’ questions, remind and monitor the patient’s use of drugs and provide regular reviews, and encourage group members to actively communicate and share nursing experiences associated with the care of patients. For patients and familied with pessimism, anxiety or depression mood, we added WeChat alone to carried out psychological counselling and psychological support one-on-one via text, voice or video way.

#### Conventional nursing

Routine medical services were provided to patients during hospitalization. The attending physician explained disease-related knowledge and surgical procedures to patients and their families. Nursing staff provided dietary knowledge, postoperative attention, methods, matters required after discharge, regular visitation and regular re-examination of coronary angiography.

### Research tools

1. SAQ: We used the SAQ with a total of 19 entries in 5 major items to evaluate the quality of life of patients [11]. The 5 items are physical activity restriction, stable angina pectoris, frequency of angina pectoris attack, satisfaction with treatment and cognitive degree of disease. Each dimension adopts the percentile system, and the higher the score, the better the quality of life.

2. SDS: Zung’s SDS was applied, which is widely used in the clinic and has high reliability and validity [12]. This scale consists of 20 items, including 10 negative symptoms and 10 positive symptoms. Each question represents a feature of depression. All items together reflect the mood, body discomfort symptoms, spiritual movement, behaviour, and psychological symptoms of patients with depression. The score involves 4 grades. The scores were obtained using the scoring method in ascending order (1 to 4) based on the occurrence frequency of the positive symptoms. The rough scores were obtained using the reverse scoring method in descending order (4 to 1) based on the occurrence frequency of the negative symptoms. The standard score was obtained by multiplying the scores by 1.25 and rounding the result. Normally, the upper limit score is 41, and the standard total score is 53. A higher score indicates a more significant depression tendency.

3. SAS: Zung’s SAS was applied, which is widely used in the clinic and has high reliability and validity [13]. Fifteen items are stated with negative words. The scores were obtained using the scoring method in ascending order (1 to 4) based on the occurrence frequency of the symptoms. Five items are stated with positive words. The scores were obtained using the reverse scoring method in descending order (4 to 1) based on the occurrence frequency of the symptoms. The total score was obtained by adding the scores of all items. The standard score was obtained by multiplying the total score by 1.25 and rounding the result. The mean value of the standard score is 50. Grade description: < 50 means normal, 50 to 59 means mild anxiety, 60 to 69 means medium anxiety, and ≥ 70 means severe anxiety.

### Statistical analysis

According to the incidence of post-discharge complications in the pre-survey, with α = 0.05, two-tailed and a power of 90%, the sample sizes of the two groups were 30 and 32, respectively, calculated by SPSS18.0. Quantitative data were expressed as the mean ± standard deviation (x ± s). The continuous data were all in accordance and normally distributed by the normal test and were statistically analyzed by the independent sample t test. Qualitative data were compared by chi-square test and Fisher’s test. A *p* value of < 0.05 was defined as statistically significant.

## Results

There were no significant differences between the two groups in general clinical data. There were also no significant differences in the scores of SAS and SDS at admission and SAQ at discharge between the two groups.(*P* > 0.05) There was no significant difference in the SAQ score between the two groups at discharge in 5 items: physical activity restriction, stable angina pectoris, frequency of angina pectoris attack, satisfaction with treatment and cognitive degree of disease.(*P* > 0.05) These results indicated that the two groups were homogeneous and comparable.

All patients were followed up for more than 1 year. One year after the operation, the SAQ score in the five items in the continuous nursing group was significantly better than that in the conventional nursing group. (*P* < 0.05) (Table 2, Table 3) The SAS and SDS scores of the continuous nursing group were significantly decreased 1 year after surgery compared to the preoperative SAS and SDS scores, and the differences were statistically significant. (*P* < 0.05) However, the SAS and SDS scores of the conventional nursing group were also lower 1 year after surgery than the scores before surgery, but these differences were not statistically significant. (*P* > 0.05) The SAS and SDS scores of the continuous nursing group were significantly lower than those of the conventional nursing group 1 year after surgery. (*P* < 0.05) (Table 4).

**Table 2:** Comparison of SAQ scores between the two groups at discharge

Item	Group A	Group B	*P* value
**Physical activity restriction**	62.5 ± 8.9	64.2 ± 10.4	0.874
**Stable angina pectoris**	54.6 ± 7.3	52.5 ± 9.6	0.912
**Frequency of angina pectoris attack**	61.3 ± 9.3	60.3 ± 11.2	0.769
**Satisfaction with treatment**	73.5 ± 10.9	75.8 ± 12.4	0.816
**Cognitive degree of disease**	63.8 ± 11.3	66.1 ± 13.2	0.743

**Table 3:** Comparison of SAQ scores between the two groups 1 year after operation

Item	Group A	Group B	*P* value
**Physical activity restriction**	83.3 ± 13.9	73.4 ± 12.5	0.032
**Stable angina pectoris**	77.6 ± 9.7	63.5 ± 8.2	0.024
**Frequency of angina pectoris attack**	80.3 ± 11.2	69.8 ± 10.5	0.018
**Satisfaction with treatment**	94.8 ± 12.3	80.3 ± 11.6	0.011
**Cognitive degree of disease**	89.5 ± 10.9	76.1 ± 12.4	0.015

**Table 4:** Comparison of psychological state before and after nursing intervention

Item	Group A	Group B	*P* value
**The SDS score**			
**Preoperation**	53.7 ± 10.8	54.3 ± 11.7	0.886
**1 year after operation**	41.3 ± 9.1*	49.2 ± 12.1	0.031
**The SAS score**			
**Preoperation**	62.3 ± 13.3	60.2 ± 15.6	0.891
**1 year after operation**	46.2 ± 10.4*	53.8 ± 12.2	0.028

The “*” indicate that compared with preoperation the *P* < 0.05

There were 2 patients with chest tightness or chest pain after discharge in the continuous nursing group and 8 patients in the conventional nursing group. There was a significant difference between the two groups.(*P* = 0.039) There were no patients with coronary restenosis after discharge in the continuous nursing group and 4 patients in the conventional nursing group. There was a significant difference between the two groups. (*P* = 0.045).

## Discussion

With the improvement of socioeconomic level, the enrichment of material objects and the arrival of an era of ageing, the incidence of coronary artery disease is increasing in recent year [14]. coronary artery bypass grafting is one of the most effective methods for treating coronary artery disease, and patients need to take long-term anti-platelet aggregation, lipid-regulating and β-receptor blocking agents to prevent the occurrence of cardiovascular adverse events. Patients often have a history of chronic diseases such as hypertension, diabetes and hyperlipidaemia and poor habits such as smoking and drinking, and the occurrence of coronary artery disease is closely related to the aforementioned chronic diseases and habits [15, 16].

Most patients underwent coronary artery bypass grafting in China are from rural areas, and their academic qualifications are generally low. The lack of disease-related knowledge, poor self-management ability, and the lack of awareness of the importance of continuous treatment result in an inability to take drugs correctly and maintain good lifestyle habits after discharge, which easily causes poor control of blood pressure, blood sugar and blood lipids, leading to adverse cardiovascular events, and even endangering the lives of patients. Some studies have shown that a deterioration of most patients’ condition after coronary artery bypass grafting is not only related to the physique of the patients themselves but also to the low compliance of drug use and the lack of postoperative nursing knowledge, ways to solve the confusion, timely medical treatment and attention to the disease [17]. After coronary artery bypass grafting, most patients die from a lack of knowledge and unhealthy lifestyle, not from the disease itself [18]. Therefore, it is essential to strengthen the patients’ management of coronary artery bypass grafting from becoming disconnected from hospitals, thus leaving the family to solve the problem of insufficient support after discharge [19]. As a result, a new nursing service model (continuous nursing) that extends treatment and rehabilitation care during hospitalization to the family to help improve the self-management of patients and ensure that information, treatment and nursing services continue without sudden interruption has been gradually applied to the clinic [20].

In this study, we found that the patients with chest tightness or chest pain and coronary artery restenosis in the continuous nursing group were significantly lower than those in the conventional nursing group 1 year after the operation. The continuous nursing group was significantly better than the conventional nursing group in terms of physical activity restriction, stable angina pectoris, frequency of angina pectoris attack, satisfaction with treatment and cognitive degree of disease. The results are suggested that continuous nursing can reduce the incidence of chest tightness or chest pain and coronary artery restenosis and improve the quality of life in patients after coronary artery bypass grafting.

For most people, surgery is a terrible shock, and there is tension and anxiety. Especially for the heart surgery, which has a high risk, the fear and tension are inevitable before and after surgery [21]. Patients underwent coronary artery bypass grafting requires taking lifelong drugs, strictly controlling blood pressure, blood glucose and blood lipids, and maintaining good lifestyle habits. For most patients, there will be a heavy psychological burden, and some patients even lose confidence and have obvious negative feelings [22, 23]. In addition, for many of the patients from rural areas, the degree of education is generally low and the knowledge about the disease is insufficient. In the short time during hospitalization, patients cannot fully learn all of the knowledge for the management of the disease from the nursing staff. The medical treatment in rural areas is inconvenient, and the problem is difficult to solve in a timely manner. Therefore, patients are more likely to be anxious and negative.

The sudden loss of nursing and professional knowledge would have a significant negative impact on the health and quality of life for the patients and families who have neither psychological adaptability nor learning how to care. Therefore, patients need more follow-up and attention in physical care and mental health care. WeChat with visibility functions is widely and conveniently used in China and it is available in a timely manner. We use WeChat as the main medium to carry out continuous nursing. The nurse instructed the patient to join the WeChat group and trained the patient on the use of the WeChat function correctly and skilfully after surgery. To increase patients’ knowledge of the disease, strengthen their self-management ability, improve their compliance, and reduce their anxiety caused by the disease, one team member was on duty every day to answer the patients’ questions, remind and monitor the patients’ on the use of drugs with regular review, and encourage group members to actively communicate and share nursing experiences associated with the care of patients. For patients with severe negative feelings and anxiety, we could also communicate as soon as possible through the WeChat platform to solve the patients’ problems in a timely manner and carry out special psychological counselling and support. At the same time, we were able to encourage patients to communicate with each other and share experiences to enhance confidence, ease negative psychological feelings and eliminate anxiety via the continuous nursing group of WeChat. In this study, the SAS and SDS scores of the continuous nursing group were significantly decreased 1 year after surgery compared to the preoperative SAS and SDS scores, and the SAS and SDS scores of the continuous nursing group were also significantly lower than those of the conventional nursing group1 year after surgery. These results suggest that continuous nursing for patients undergoing coronary artery bypass grafting can significantly alleviate depression and anxiety.

There are several limitations in this study. First, this study was a retrospective study with a small sample size, and patients who did not have independent reading ability, communication equipment or convenient information communication were not included in this study, which may cause selection bias. Second, this study was a single-centre study, and more research from multiple centres is mandatory to assess the value of continuous nursing in the future. Third, the follow-up period of this study was brief, and a longer term follow-up period is needed.

## Conclusion

Continuous nursing extends high-quality hospital nursing service and psychological support to the patient’s family, which improved patient compliance with treatment, reduced the occurrence of postoperative complications, and provided the patient with proper psychological evaluation to relieve anxiety and depression and improve their quality of life.

## Data Availability

Data sharing not applicable to this article as no data sets were generated or analyzed during the current study.

## Abbreviations

SDS: Self-Rating Depression Scale
SAS: Self-Rating Anxiety Scale
SAQ: Seattle Angina Pectoris Questionnaire
